# Synthesis of Different Layers of Graphene on Stainless Steel Using the CVD Method

**DOI:** 10.1186/s11671-016-1709-x

**Published:** 2016-11-16

**Authors:** Ferial Ghaemi, Luqman Chuah Abdullah, Paridah Md Tahir, Robiah Yunus

**Affiliations:** 1 Institute of Tropical Forestry and Forest Products (INTROP), Universiti Putra Malaysia (UPM), 43400, Serdang, Selangor Malaysia; 2 Department of Chemical and Environmental Engineering, Universiti Putra Malaysia (UPM), 43400, Serdang, Selangor Malaysia

**Keywords:** Graphene, Stainless steel, Chemical vapor deposition

## Abstract

In this study, different types of graphene, including single-, few-, and multi-layer graphene, were grown on a stainless steel (SS) mesh coated with Cu catalyst by using the chemical vapor deposition (CVD) method. Even though the SS mesh consisted of different types of metals, such as Fe, Ni, and Cr, which can also be used as catalysts, the reason for coating Cu catalyst on the SS surface had been related to the nature of the Cu, which promotes the growth of graphene with high quality and quantity at low temperature and time. The reaction temperature and run time, as the most important parameters of the CVD method, were varied, and thus led to the synthesis of different layers of graphene. Moreover, the presence of single-, few-, and multi-layer graphene was confirmed by employing two techniques, namely transmission electron microscopy (TEM) and Raman spectroscopy. On top of that, electron dispersive X-ray (EDX) was further applied to establish the influence of the CVD parameters on the growth of graphene.

## Background

Graphene is one of the most interesting nanomaterials applied in many expanding disciplines of contemporary studies. This nanomaterial, a two-dimensional (2D) material, is formed by sp^2^-bonded carbon atoms that are highly packed into a 2D honeycomb lattice that owns excellent characteristics, such as mechanical, thermal, and electrical properties [1, 2]. In fact, since 2004, many methods have been improved to fabricate high quality and quantity of graphene materials [3–6]. Graphene is classified into three types, including single- or double-layered graphene (SLG; about 1–2 layers), few-layered graphene (FLG; consisting of 3–5 layers), and multi-layered graphene (MLG; consisting of 5–10 layers) [7, 8].

Since decades ago, coating carbonic materials on steel in the steel industry has been carried out to improve the steel properties by using of a low amount of carbon materials [9]. Among the different types of carbonic materials such as amorphous carbon materials, graphene with unique properties offers highly promising opportunities for the development of stainless steel surface not only to prevent electrochemical corrosion but also has potential applications in nano-electronics, molecular separation, high-strength composites, and surface coating industries [10–12].

In this process, steel is placed into a carbon-rich environment at high temperatures for a certain amount of time and then quenched. Although the processes for coating the metal with carbonic material are different from the rest, the chemical vapor deposition (CVD) technique is generally used to grow graphene on the surface of the metals. Therefore, CVD, as one of the most frequently used techniques, was applied to synthesize graphene [13, 14]. Furthermore, a few researchers did probe into synthesizing graphene on stainless steel (SS) or iron substrate. Though the yield of the graphene produced through CVD method is very poor, the quality of the obtained graphene is high [15, 16]. On the other hand, Cu has a main role to synthesize graphene in a large scale [17]. Therefore, in this research, in order to overwhelm the drawback of producing a large-scale amount of graphene and also keep its quality, CVD on Cu (as promoter catalyst) is considered as one of the most promising methods due to its fabrication in a large-scale and high-quality graphene [18, 19].

In addition, to achieve the different layers of graphene (SLG, FLG, and MLG) on stainless steel substrates, some parameters of the CVD method should be optimized. Therefore, the most important parameters of the CVD technique, such as reaction temperature and time, which affected the graphitization, the structures, and the amount of the different layers of graphene on the SS surface, had been changed. Hence, by controlling these two parameters, the number of graphene layers changed as well. Besides, the SS surface was coated with Cu solution, which had never been attempted before, to promote high quality of graphene sheet on SS surface. On top of that, some analytical instruments were applied to evaluate the resulting graphene. Raman spectroscopy, as a non-invasive method, was utilized to determine the number of graphene layers and graphitization [20]. Other than that, electron microscopes, such as scanning electron microscopy (SEM) and transmission electron microscopy (TEM), were used to determine the morphology and the structure of the different types of graphene on the SS. Moreover, electron dispersive X-ray (EDX) was employed to investigate the composition of the products.

## Methods

In this work, stainless steel (150 meshes, NE Scientific Co., Malaysia) that comprised of 50-μm diameter fibers was purchased and applied as a substrate to synthesize the graphitic structures on its surface. The composition of the SS mesh was obtained by using EDX and is given in the following: C 0 wt%, Si 0.53 wt%, Cr 19.10 wt%, Ni 8.6 wt%, and Fe 70.3 wt%, while the rest had been related to other components. The pristine SS mesh was cut into a circle shape with 2-cm diameter and immersed into 9 M sulfuric acid solution for a few minutes to pretreat the SS surface. After that, the SS mesh was immersed into 50 mM Cu(NO_3_)_2_.3H_2_O solution and sonicated for 2 h to coat the surface of SS with Cu particles. The EDX results of SS mesh coated with Cu are as follows: C 0%, O 5.3%, Si 0.21%, Cr 10.2%, Ni 5.2%, Fe 46.2%, and Cu 32.2%, while the remaining weight was related to other components. The presence of oxygen was associated with the elemental composition of Cu nitrate trihydrate. Besides, when the SS mesh was placed into the CVD reactor and the temperature was increased to 300 °C, the nitrate compound was removed.

In order to synthesize the graphene, pure acetylene (C_2_H_2_) was used as the hydrocarbon source, while nitrogen (N_2_) and hydrogen (H_2_) were utilized as the carrier gases. Next, the reaction temperature and the time in the CVD technique were altered to grow the graphene with different layers. First, the SS mesh was located in the CVD chamber, and then, the temperature was increased (950, 1000, and 1050 °C) under the flow of N_2_ gas. When the system reached the required temperature, the acetylene gas as a carbon source was inserted into the system at 50 sccm with the presence of H_2_/N_2_ flow (10 ssm/50 sccm), for different run times from 10 to 50 min. Finally, the C_2_H_2_ flow rate was stopped, and the furnace was turned off under the presence of N_2_ flow. Thus, in order to evaluate the morphology, graphitization and composition of the produced nanomaterials, Raman spectroscopy, SEM, TEM, and EDX were employed.

## Results and Discussion

**Fig. 1:**
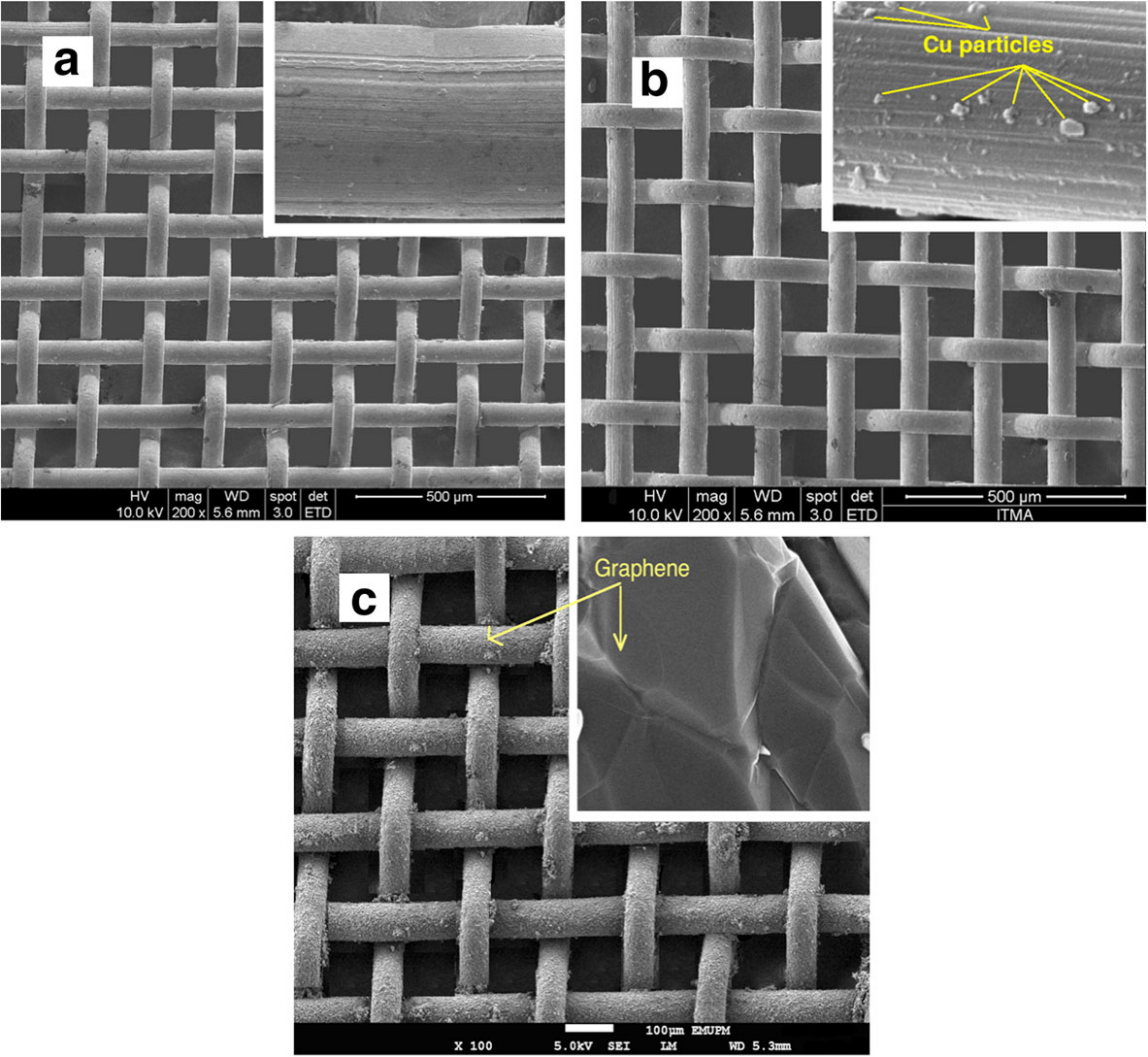
SEM images of **a** neat SS mesh, **b** Cu particles on SS, and **c** graphene-coated SS

In this research, different layers of graphitic materials were produced on the SS mesh via CVD technique. Hence, in attaining this aim, reaction temperature and run time had to be optimized to control the number of graphene layers. SEM images in Fig. 1 illustrate the neat SS mesh and the SS coated with graphene.

### Optimization of the CVD Parameters

The reaction temperature and the run time, as the most impotent parameters of the CVD method, were altered in order to determine the optimum condition to produce SLG, FLG, and MLG on the SS mesh. To further evaluate the effects of the reaction temperature and the run time on both the structure and the morphology of the grown graphene on the SS mesh, Raman spectroscopy, TEM, and EDX were applied.

#### Temperature Effects

**Fig. 2:**
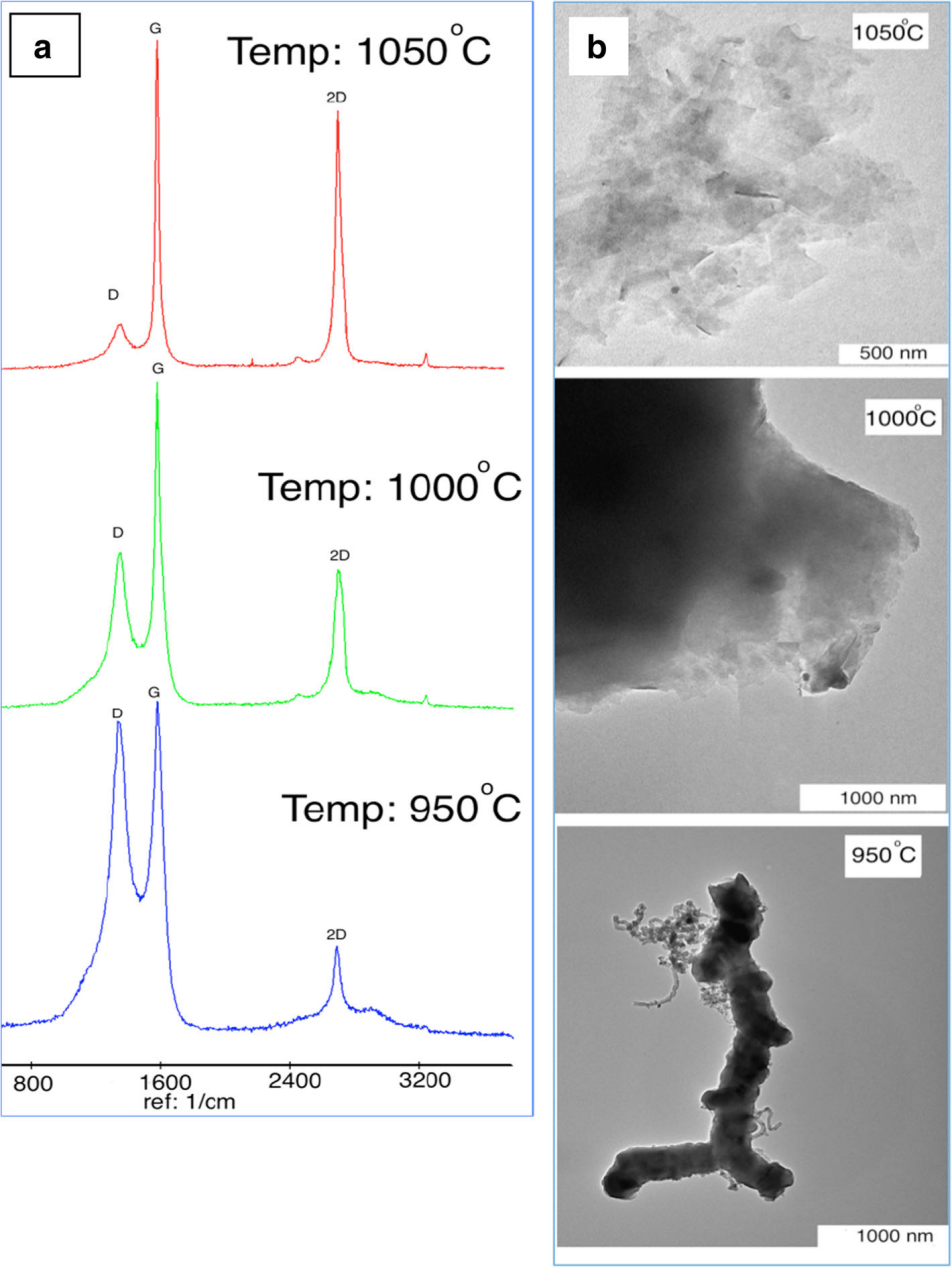
**a** Raman spectra and **b** TEM images of graphitic structures at different temperatures

To study the influence of the reaction temperature on the structure and the morphology of the products, the temperature was increased from 950 to 1050 °C at 30 min run time under 50/50/10 sccm with C_2_H_2_/N_2_/H_2_ flow rate. The number of graphene layers and also the graphitic structures were determined by using the Raman spectroscopy, which is not only a non-destructive method but also a fast one [21]. In the Raman spectra, D peak (~1350 cm^−1^) and 2D peak (~2650 cm^−1^), in relation to the electronic and geometrical structures through the double-resonance process, were investigated. The D peak provided information about the breathing modes of the sp^2^ atom [22] activated by the presence of any defective structure (e.g., lattice disorder, edges, or functional groups [23]). Another peak was the G peak (~1580 cm^−1^), which was associated with an E_2g_ stretching mode of the graphitic structure. The stretching of the C–C bond in graphitic materials gave rise to the G peak, which was related to all sp^2^ carbon systems. Moreover, the intensity ratio of the D peak and the G peak (I_D_/I_G_) was utilized to evaluate the degree of graphitization [23]. Besides, the high amount of the I_2D_/I_G_ ratio revealed the high quality and also the low number of graphene layers [24]. Therefore, by increasing the I_2D_/I_G_ ratio, the number of graphene layers decreased. The Raman spectra at different temperatures are displayed in Fig. 2a. It had been discovered that by increasing the temperature from 950 to 1050 °C, the intensity ratio of I_D_/I_G_ decreased, but I_2D_/I_G_ increased, which showed that the graphitization grew with the decrease in the number of graphene layers (increased 2D peak and decreased D peak).

Moreover, the TEM images in Fig. 2b revealed the morphology and the structures of the produced materials. In order to carry out the TEM test, the SS mesh was immersed into acetone 60% solution and agitated ultrasonically for 15 min to separate the graphene sheet from the SS surfaces. Based on the achieved TEM images, the carbon nanofiber and the carbon nanotubes were produced at 950 °C, but when the temperature reached 1000 °C, the multi-layer graphene was synthesized and the number of graphene layer decreased to few layer at 1050 °C. Besides, based on the EDX results, by increasing the temperature, the carbon atoms increased (Table 1). Therefore, it was found that by increasing the temperature, the amount of graphene increased, but the number of graphene layers decreased.

**Table 1:** EDX results at different reaction temperature

Component (wt%)	950 °C	1000 °C	1050 °C
C	18.2	22.6	24.8
O	4.1	3.9	4.2
Cr	10.1	9.2	8.7
Ni	4.8	4.2	4
Fe	32.1	30	28.7
Cu	30.1	29.6	29.2
Others	Rest	Rest	Rest

#### Time Effects

Reaction time leads to the activation of the metallic catalyst to grow the different number of layers of graphene with high quality and graphitization. Therefore, the time had to be optimized for each type of graphitic structures by using different times (10, 30, and 50 min) while the other parameters were kept constant (at 1050 °C under N_2_/H_2_/C_2_H_2_ flow rates for 50/10/50 sccm) to determine the effects of the reaction time on the graphitization, as well as the morphology of the products.

**Fig. 3:**
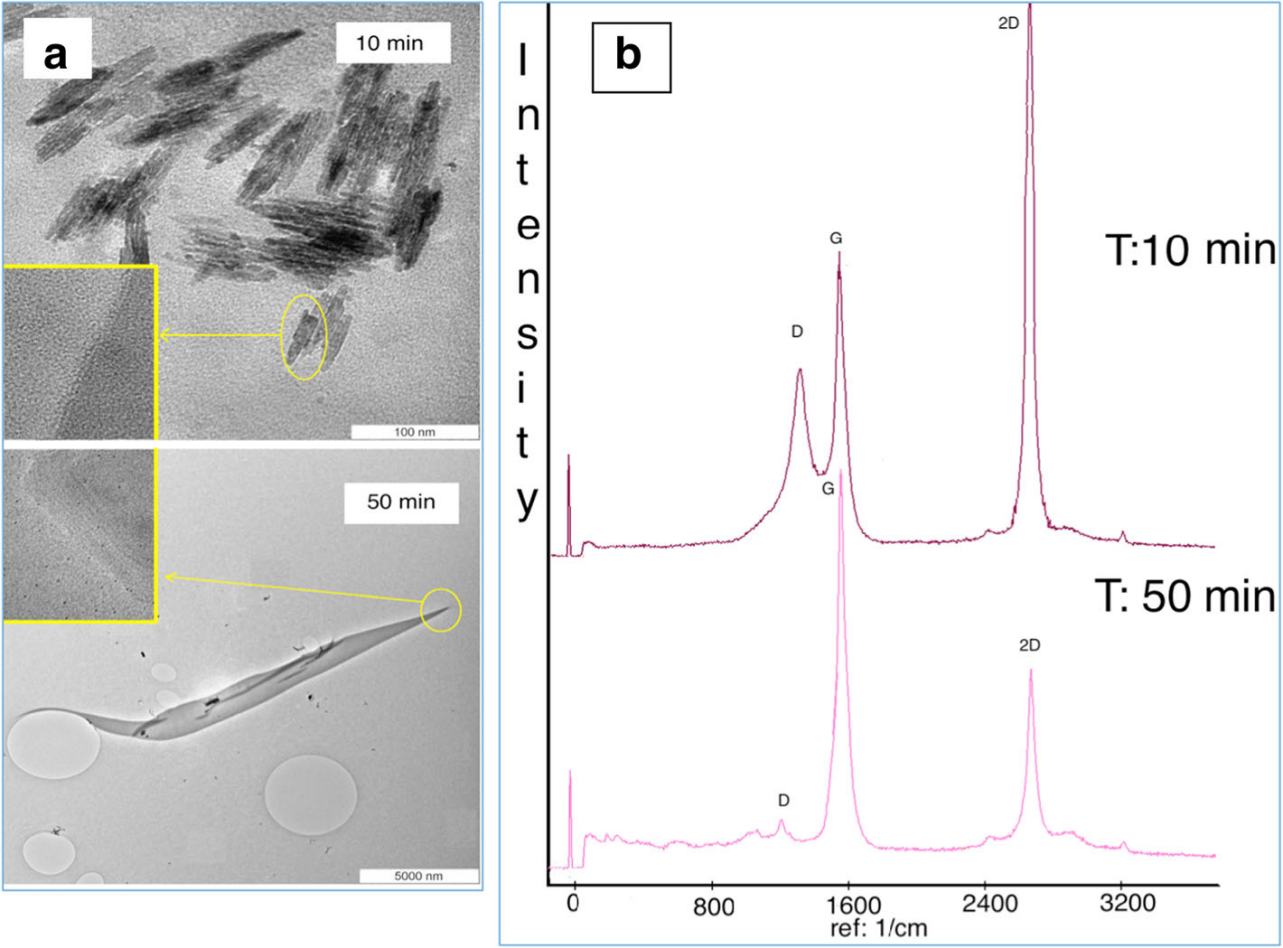
**a** Raman spectra and **b** TEM images of graphitic structures at different time

Based on the Raman spectroscopy, it was found that by changing the reaction time, the intensity of the 2D and D peaks of the Raman spectra altered significantly (Fig. 3). So, by increasing the reaction time, the number of graphene layers and the amount of graphene that coated the SS surface increased, as proven by the low intensity of the 2D and D peaks, respectively (see Fig. 3). At the 10th min, however, single layers of graphene were produced on the SS surface (I_2D_/I_G_ ratio was about 2) [25], which was insufficient to completely coat the SS surface with graphene. Hence, some parts of the SS surface were coated with SLG, whereas some parts were uncovered. This phenomenon was confirmed by the high intensity of D peak. Contrarily, at the 50th min, the number of graphene layers increased and successfully covered the SS surface completely. Besides, the results obtained from the EDX proved that by increasing the time, the amount of the carbon atoms increased (Table 2). Therefore, by analyzing the trend of the number of graphene layers from Raman spectra and the amount of C atoms from EDX, it was found that by increasing the time from 10 to 50 min, the number of graphene layers and the amount of C atoms experienced an increase. In conclusion, the reaction time of 10 min was suitable for growing single-layer graphene, while 30 min was the optimum reaction time to synthesize a few layers of graphene and 50 min to grow multi-layer graphene on the SS at 1050 °C.

**Table 2:** EDX results at different reaction time

Element (wt.%)	10 min	50 min
C	10.2	27.8
O	4.6	3.9
Cr	9.1	7.9
Ni	4.6	3.7
Fe	40.7	27.6
Cu	30.2	28.8
Others	Rest	Rest

## Conclusions

The most important goal of this research was to grow different layers of graphene on the SS mesh. Hence, a simple method was employed to fabricate different layers of graphene sheets on SS surface coated with Cu particles as a promoting catalyst for graphene growth in a large scale with high quality via CVD technique. Therefore, in order to determine the different layers of graphene, some parameters of the CVD process had been changed. The reaction temperature was ranged from 950 to 1050 °C, while the reaction time was varied from 10 to 50 min. On the other hand, the effects of these parameters on the graphitization, the number of graphene layers, and the amount of C atoms of the product were demonstrated by Raman spectroscopy and EDX, respectively. In addition, SEM and TEM were used to analyze the morphology and the structures of the graphene on SS mesh. Based on the results, single-layer graphene on SS with about 10.2 wt.% C atoms was grown at 1050 °C for 10 min under the 50 sccm acetylene flow rate with low graphitization (I_D_/I_G_ = 0.4 and I_2D_/I_G_ = 1.94). Meanwhile, the FLG with 24.8 wt.% C atoms was grown at 1050 °C for 30 min under the 50 sccm acetylene flow rate with high graphitization (I_D_/I_G_ = 0.1 and I_2D_/I_G_ = 0.75). Other than that, the MLG with about 22.6 wt.% C atoms was synthesized at 1000 °C for 30 min under the 50 sccm acetylene flow rate with low graphitization (I_D_/I_G_ = 0.5 and I_2D_/I_G_ = 0.45). Furthermore, by increasing the temperature to 1050 °C for 50 min, the MLG was achieved with 27.8 wt.% C atoms and the highest graphitization was attained (I_D_/I_G_ = 0.05 and I_2D_/I_G_ = 0.5). Nonetheless, it is worth noting that the growing of the structures with low graphitization at 950 °C for 30 min under the 50 sccm was related to non-graphene materials (I_D/IG_ = 0.95 and I_2D/IG_ = 0.24).

## Abbreviations

CVD: Chemical vapor deposition
EDX: Electron dispersive X-ray
FLG: Few layer graphene
MLG: Multi-layer graphene
SEM: Scanning electron microscopy
SLG: Single-layer graphene
SS: Stainless steel
TEM: Transmission electron microscopy
